# Contribution of pilus type 2b to invasive disease caused by a *Streptococcus agalactiae* ST-17 strain

**DOI:** 10.1186/s12866-017-1057-8

**Published:** 2017-07-03

**Authors:** Maddalena Lazzarin, Rong Mu, Monica Fabbrini, Claudia Ghezzo, C. Daniela Rinaudo, Kelly S. Doran, Immaculada Margarit

**Affiliations:** 1 GSK S.r.l., Siena, Italy; 2 Department of Biology and Center for Microbial Sciences, San Diego State University, 5500 Campanile Dr., NLS 317, San Diego, CA 92182 USA; 3 Department of Pediatrics, University of California San Diego School of Medicine, La Jolla, CA 92093 USA

**Keywords:** GBS, *Streptococcus agalactiae*, Pilus protein, Pilus island, Mouse meningitis model, Host cell adherence, Host cell invasion

## Abstract

**Background:**

Group B Streptococcus (GBS) is a major cause of invasive disease especially in neonates. In GBS three structurally distinct pilus polymers have been identified as important virulence factors and promising vaccine candidates. The vast majority of Group B Streptococci belonging to the hypervirulent serotype III ST-17 lineage bear pilus types 1 and 2b. The purpose of this study was to investigate the relative contribution of these two pilus types to the pathogenesis of a ST-17 strain.

**Results:**

We performed in vivo and in vitro analysis of isogenic knockout mutants derived from the GBS COH1 ST-17 strain deprived of either pilus type 1 or 2b. We compared the two pilus mutants with the wild type strain in a mouse model of invasive disease, in vitro survival in macrophages, and adherence/invasion assays using human brain endothelial and lung epithelial cell lines. Significantly less of the pilus 2b mutant was recovered from the blood, lungs and brain tissue of infected mice compared to the wild-type and pilus 1 mutant strains. Further, while the pilus 2b mutant survived similarly in murine macrophages, it exhibited a lower capacity to adhere and invade human brain epithelial and lung endothelial cell lines.

**Conclusions:**

The data suggest an important role of pilus 2b in mediating GBS infection and host cell interaction of strains belonging to the hypervirulent GBS ST-17 lineage.

## Background

*Streptococcus agalactiae* (also known as Group B *Streptococcus*, GBS) colonizes asymptomatically the gastrointestinal and genitourinary tract of up to 30% of healthy adults and can cause serious illness in neonates, the elderly and imuno-compromised patients [1, 2]. Neonatal infection mainly occurs between 0 and 6 days of life (early onset disease, EOD) and up to 3 months (late onset disease, LOD). Most common disease manifestations are sepsis, pneumonia and/or meningitis. EOD incidence has declined after implementation of intrapartum antibiotic prophylaxis [3, 4]. However, this practice has failed to reduce LOD and has raised concerns regarding antibiotic resistance and allergies, renewing the interest in GBS vaccines [5, 6].

GBS is classified into ten capsular polysaccharide serotypes and several sequence types (ST), and the serotype III is the most frequent among neonatal disease isolates [7]. In particular, type III strains belonging to the hypervirulent Clonal Complex 17 (CC17 and highly related sequence types) have been significantly associated with meningitis and account for up to 44 EOD and 67% LOD cases compared with less than 10% of colonizing isolates [8, 9].

GBS expresses several virulence factors mediating adherence and invasion of host cells, penetration of epithelial/endothelial barriers, and evasion from the innate immune system. Among these are the cell wall-anchored pilus polymers that protrude outside the bacterial surface and are constituted by covalently linked subunits, i.e. the backbone protein (BP) and the ancillary proteins (AP), AP1 and AP2 located at the tip and the base of the structure, respectively [10]. The BP and AP1 proteins were identified for their protective capacity against GBS infection in a mouse neonatal challenge model [11].

Three pilus variants (type 1, 2a and 2b) were described in GBS, and all strains carry at least one variant [11]. A relationship was observed between the presence of particular GBS pilus type profiles and the different serotypes and phylogenetic lineages. The vast majority of hypervirulent CC17 isolates contain pilus 1 plus pilus 2b genes, while this combination is rare among other clonal complexes [12]. Few human-derived ST-17 strains that have lost the genomic locus coding for the pilus 1 have been recently reported [13, 14].

The contribution of pili to GBS pathogenesis was initially investigated on strains expressing the pilus 2a; decreased adherence and invasion to human epithelial or endothelial cell lines was observed for mutants lacking this pilus type [15, 16]. More recently, the AP1-2a protein has been shown to mediate neutrophil recruitment, enhanced penetration of the blood-brain barrier (BBB) and meningitis in a mouse infection model [17]. More controversial results have been obtained for pilus 1, as some authors reported a role in cell adherence and transcytosis and others in cell invasion and survival inside the macrophages but not in cell adhesion [18]*.* Decreased invasion of several host cell types [19] and survival inside macrophages [20] have also been reported for a knockout mutant of the pilus 2b backbone protein.

The phenotypes of some pilus knockout mutants were recently shown to differ depending on the strain background [21]. Furthermore, the relative contribution of GBS pilus 1 and pilus 2b to GBS infection has not been investigated in vivo. In the present work, the phenotypes of isogenic knockout mutants of a ST-17 strain deprived of BP-1 or BP-2b proteins were compared with the parental wild-type (WT) strain in a mouse bacteremia/meningitis model, for survival inside the phagocytes and in cell-based adhesion and invasion assays.

## Results

### Generation of knockout mutants unable to express pili 1 or 2b

To study the role of pili 1 and 2b in the pathogenesis of the GBS COH1 ST-17 strain that highly expresses both types of pili [10] we analyzed two knockout (KO) mutant derivatives deprived of the backbone protein genes. The COH1 KO mutant *Δbp-1* lacking pilus 1 backbone protein (BP-1) (also named Δ80) was previously obtained and shown to be unable to assemble pilus 1 polymers [10]. In the same COH1 background, we generated a second mutant deleted of the gene coding for the backbone protein of pilus 2b (BP-2b). This KO mutation was complemented by a plasmid expressing the wild type gene (pAM_*bp-2b*).

**Fig. 1:**
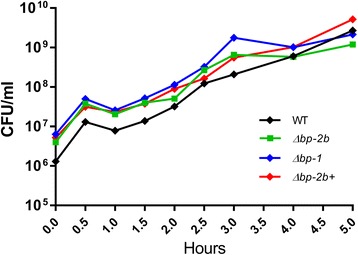
The pilus 2b mutant (Δ*bp-2b*) and its complemented derivative (Δ*bp-2b+)* show equivalent growth in rich medium to wild-type (WT) and the pilus 1 mutant (Δ*bp-1*). GBS bacteria were inoculated in Todd Hewitt Broth (THB) and incubated at 37 °C. The number of viable bacteria was assessed at different points up to 5 h. Each GBS strain was run in triplicate for each time point and the mean of triplicates ± the SD are shown. No significant differences were observed among strains (*P* > 0.05, one-way ANOVA non parametric test followed by Kruskal-Wallis test)

Growth kinetics in rich medium of the pilus 2b mutant and its complemented strain were equivalent to those of GBS COH1 wild-type and the *Δbp-1* mutant (Fig. 1, all *P* > 0.05, Kruskal-Wallis test).

**Fig. 2:**
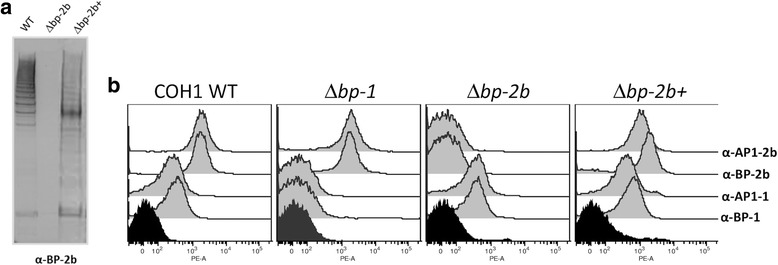
Detection of pilus 2b polymerization and bacterial surface expression of pilus proteins. **a** Western blot analysis of total proteins from wild-type COH1 (WT), knockout mutant strain (Δ*bp-2b*) lacking pilus 2b backbone protein (BP-2b) and Δ*bp-2b* strain complemented with a plasmid expressing BP-2b (Δ*bp-2b+*). The membrane was probed with a mouse monoclonal antibody (mAb) raised against BP-2b (α-BP-2b). **b** Flow cytometry analysis on whole fixed bacteria corresponding to wild-type COH1, mutant COH1 strains lacking the backbone protein of pilus 1 (Δ*bp-1*) or of pilus 2b (Δ*bp-2b*) and to the complemented strain Δ*bp-2b+*. Bacteria were probed with mAbs against the backbone and the major ancillary proteins of pilus 1 (*α* -BP-1 and AP1–1) and pilus 2b (α -BP-2b and AP1-2b) and labeled with R-Phycoerythrin conjugated goat anti-mouse secondary antibodies. Black histograms indicate staining of bacteria with secondary antibodies only. The data are representative of three independent experiments

Total proteins from the native COH1 strain, its *Δbp-2b* mutant and the complemented derivative (*Δbp-2b+)* were analyzed by Western Blot using a monoclonal antibody against the BP-2b. WT extracts revealed the typical high-molecular-weight ladder indicative of pilus structures, whereas this ladder was not present in the *Δbp-2b* mutant strain; complementation of the KO strain restored pilus 2b expression (Fig. 2a).

Surface expression of BP and AP1 proteins of pilus 1 and 2b on WT, isogenic mutants and *Δbp-2b +* was assessed by Flow Cytometry using whole bacteria. Comparable fluorescent signals against BP-1 were observed for the WT and *Δbp-2b* strains, while *Δbp-1* showed no signal. Fluorescent signals against BP-2b were similar for WT and *Δbp-1* strains and absent in *Δbp-2b* strain (Fig. 2b). These data confirmed that deletion of the gene encoding the backbone protein from each pilus island prevents the formation of the corresponding pilus polymers and does not affect the expression of the other island. Fig. 2b also shows that the both AP1–1 and AP1-2b proteins were undetectable on the surface of the corresponding BP mutants, while mutation of the heterologous BP had no effect on their surface exposure.

### The pilus 2b contributes to bacteremia and penetration of the blood-brain barrier

**Fig. 3:**
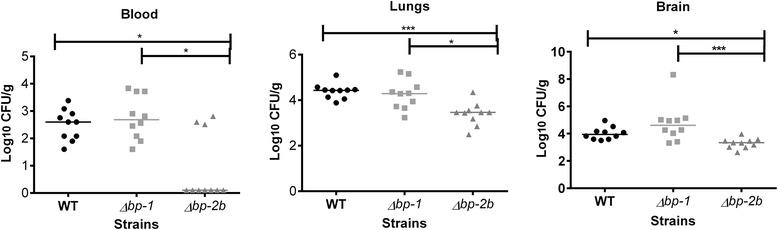
Type 2b pili contribute to infection in vivo by ST-17 COH1. Ten CD-1 mice (8-week-old) for each group were injected via tail vein with 1.5 × 10^8^ Colony Forming Units (CFU) of wild-type COH1 (WT), Δ*bp-1* or Δ*bp-2b* mutants. Blood, brains and lungs were collected upon euthanasia at day 2 post-infection, the tissues were homogenized and plated for enumeration of bacterial CFU. Horizontal lines represent median CFU values. Differences between groups were analyzed by Kruskal-Wallis test followed by Dunn’s multiple comparisons test. Only statistically significant differences are reported (*P < 0.05, **P < 0.01, ***P < 0.001)

The contribution of pilus 1 and 2b to GBS COH1 infection in vivo was investigated using a mouse model of hematogenous meningitis. Groups of 10 CD1 mice were intravenously injected with WT, *Δbp1* or *Δbp-2b* GBS bacteria (1.2 × 10^8^ CFU, 1.4 × 10^8^ CFU and 1.8 × 10^8^ CFU respectively). Mice were monitored and euthanized after 48 h and bacteria were counted in blood, lungs and brain homogenates. As shown in Fig. 3, the number of bacteria in the blood and tissues of wild-type and *Δbp-1* -infected mice groups were equivalent (all *P* > 0.99, Kruskal-Wallis followed by Dunn’s test). Conversely, significantly lower *Δbp-2b* CFU compared to the WT were detected in the blood (*P* < 0.05), lungs (*P* < 0.001) and brain (*P* < 0.02) of infected mice. The *Δbp-2b* mutant also showed decreased infectivity compared to the *Δbp-1* strain, with lower bacterial loads in blood (*P* < 0.01), lungs (*P* < 0.02) and brains (*P* < 0.001).

**Fig. 4:**
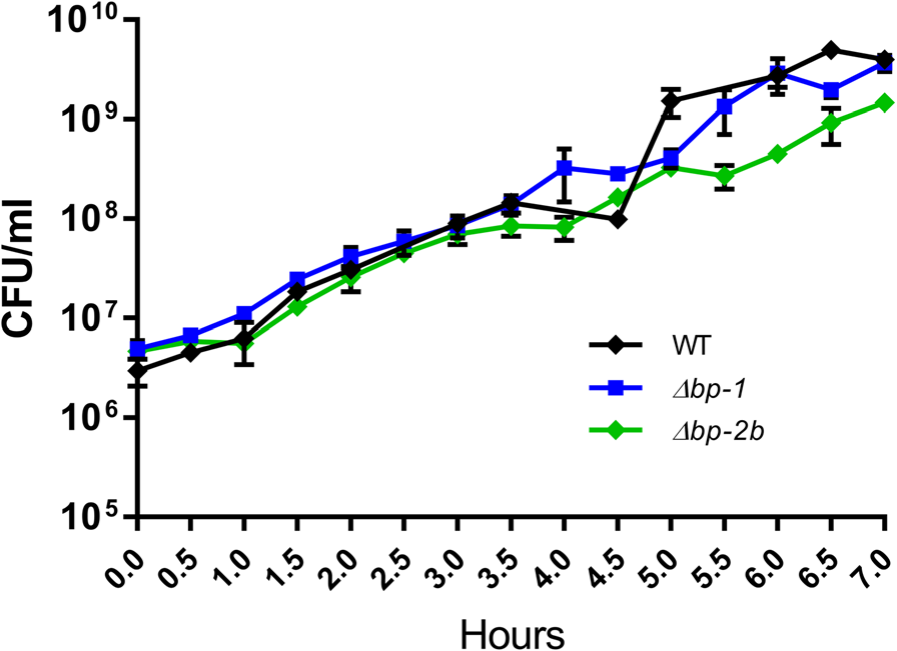
Wild-type and pilus mutant COH1 strains have equivalent growth kinetics in murine blood. In vitro blood survival assays were performed with mid log phase GBS corresponding to wild-type COH1 (WT) and mutant COH1 strains lacking the backbone protein of pilus 1 (Δ*bp-1*) or of pilus 2b (Δ*bp-2b*). Bacteria were added to mice fresh blood and incubated at 37 °C. The number of viable bacteria was assessed every 30 min up to 7 h. Each GBS strain was run in triplicate for each time point. No significant differences could be observed among the tested strains

**Fig. 5:**
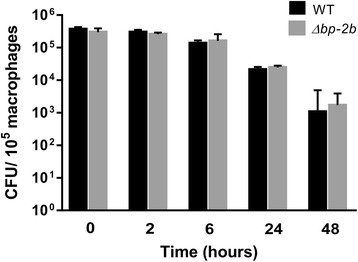
Wild-type COH1 and its pilus 2b mutant strain show equivalent phagocytosis and intracellular survival in murine macrophages. Monolayers of J774A.1 murine macrophages were incubated for 2 h with wild-type GBS COH1 or its Δ*bp-2b* mutant (MOI 10:1). After infection, cultures were incubated in presence of antibiotics for up to 48 h. At time points 0, 2 h, 6 h, 24 h and 48 h cells were lysed and plated for bacterial quantification. Data are presented as the mean CFU +/− SD from two independent experiments performed in quadruplicate. No statistical differences between strains were observed (Mann-Withney test)

To investigate whether the in vivo attenuated phenotype of *Δbp-2b* was associated to a decreased capacity of these bacteria to evade innate immune defenses, GBS WT and the two mutant strains *Δbp-1* and *Δbp-2b* were inoculated in mouse blood (2 × 10^4^/ml) and bacterial growth was followed for 7 h. As shown in Fig. 4, both mutants exhibited equivalent growth kinetics in blood to the WT parent strain (all *P*-values > 0.05, two-way ANOVA test). These data suggested that the presence of pilus 2b in GBS COH1 did not mediate bacterial protection from phagocytic killing. To further investigate this topic, we conducted non-opsonic phagocytosis and survival experiments using murine macrophages. Wild-type and *Δbp-2b* bacteria were incubated for 2 h with the J774 macrophage cell line (MOI 10:1), followed by antibiotic treatment to eliminate cell-adhered bacteria. GBS CFU were enumerated from washed cell lysates immediately after antibiotic exposure and 2, 6, 24 and 48 h later. The results reported in Fig. 5 showed equivalent numbers of cell associated bacteria at all tested time points (all *P* > 0.05, Mann Withney test). Overall, the obtained data indicated a lower survival of GBS *Δbp-2b* during in vivo infection compared to the wild-type and *Δbp-1* strains, and that differences of bacterial loads found in blood and tissues from infected mice were not due to a growth defect or to a decreased capacity of the pilus 2b mutant to survive inside live phagocytes.

### The pilus 2b contributes to GBS adherence and invasion of host cells

**Fig. 6:**
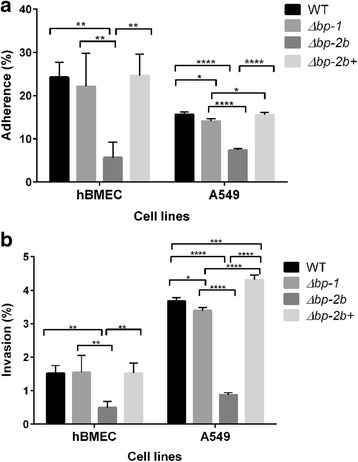
Role of type 2b pili in GBS ST-17 COH1 host cell adherence and invasion. **a** Adherence and **b** invasion assays were performed in hBMEC and A549 cells with COH1 WT and isogenic mutants. Mid-log phase bacteria were added to confluent cell monolayers at a multiplicity of infection of 1. Total cell-associated GBS bacteria were recovered after 30 min, and intracellular bacteria were recovered after 2-h infection plus 2-h incubation with antibiotics as described in Materials and Methods. GBS was quantified by serial dilution plating to enumerate CFU and expressed as (recovered CFU/initial inoculum CFU) ×100%. All adherence and invasion assays were performed in triplicate and repeated at least three times. Each bar represents means and standard deviation of three independent experiments, each performed in triplicate. Differences between groups were analyzed by One-Way ANOVA followed by Tukey’s multiple comparisons test. Only statistically significant differences are reported (* *P* < 0.05, ** *P* < 0.01, *** *P* < 0.001, **** *P* < 0.0001)

We subsequently investigated whether the lower bacterial loads in mice infected with *Δbp-2b* could be associated with a reduced ability of the mutant to adhere to or invade host epithelial and endothelial cells. To this aim, we compared adherence and invasion of human brain microvascular endothelial cells (hBMEC) and lung epithelial cells (A549) by COH1 WT, its pilus 1 and pilus 2b KO derivatives and the *Δbp-2b* complemented mutant (Fig. 6). Mid-log grown bacteria were added to confluent cell monolayers at a MOI of 1 and cell-associated GBS were plated for CFU counting after 30 min of incubation (cell adherence plus short time invasion) or after 2 additional hours of infection plus 2 h with antibiotics (cell invasion).

Of the two mutant strains, the *Δbp-1* presented comparable levels of brain endothelial cell adhesion and invasion to the wild-type strain (all *P* > 0.9, ONE-way ANOVA followed by Tukey’s multiple comparisons test), and less than 2 fold difference using epithelial pulmonary cells (*P* < 0.05) (Fig. 6a and b). Conversely, as also shown in Fig. 6a and b the *Δbp-2b* showed significantly less adherence and less invasive capacity than the wild-type strain and the *Δbp-1* both in endothelial hBMEC (*P* < 0.01) and lung epithelial cells (*P* < 0.0001). The pilus 2b complemented strain (*Δbp-*2b+) restored similar or higher levels of adherence and invasion relative to WT and *Δbp-1* strains.

## Discussion

The relevance of pili in GBS virulence has been demonstrated in several studies where the pilin subunits were shown to mediate initial bacterial attachment, invasion and transcytosis of host cells, and to enhance GBS infection in animal models [15–18]. Most studies have concentrated on pilus types 1 or 2a, while the type 2b pilus remained less characterized from a functional point of view [19–21]. Our findings demonstrate for the first time that pilus 2b significantly contributes to infection and BBB penetration in vivo in a strain belonging to the hypervirulent lineage ST-17, leading to sepsis and meningitis development. In the mouse model, bacterial counts in blood and tissues from mice injected with the pilus 2b knockout mutant were significantly lower in comparison with those from mice injected with the WT or the *Δbp-1* mutant strain.

Recent genomic studies have shown that the hypervirulent CC17 shows high homogeneity with an extremely low recombination rate relative to the other CCs [22]. Still some non-core genome differences have been reported [13, 14]. Whether the in vivo attenuated phenotype of the pilus 2b mutant observed for the COH1 strain also applies to other CC17 isolates will deserve further investigation.

Chattopadhyay and colleagues compared another serotype III ST-17 strain (GBS 874391, RDP III-3) with its isogenic pilus 2b–deficient mutant and observed 1.5–2 fold decreased intracellular survival in the J774 murine macrophage cell line [20]. We could not ascribe the reduced in vivo fitness of the GBS COH1 strain pilus 2b mutant to a higher sensitivity to phagocytes, as no differences in the growth in murine blood or in survival inside macrophages were observed between the wild-type and the mutant strain.

Conversely, we observed that the *Δbp-2b* mutant exhibited a decreased ability to adhere to and to invade brain endothelial and lung epithelial cells in comparison to both the WT and *Δbp-1* strains. Decreased capacities of pilus 2b mutants to invade a variety of host epithelial cells were formerly reported for GBS 874391 [19] and for another GBS III strain [21]. It is worth noting that adherence and invasion were not completely abolished in the COH1 pilus 2b mutant or the formerly investigated type III strains [19, 21]. Similarly, even though the number of *Δbp-2b* CFU was lower relative to the wild-type, mutant bacteria were still present in infected mice and especially in their lungs and brain. The data confirm that GBS pathogenesis is the result of the interplay between different virulence factors. This could in part explain the reported differences in the impact of pilus deletions on different background strains.

Flow cytometry analysis of the *Δbp-2b* mutant confirmed the complete absence of BP-2b on the bacterial surface, and revealed that the major pilus ancillary protein AP1 was also undetectable in this mutant. The AP1 protein of GBS pilus type 2a and the corresponding ancillary protein from other Gram positive bacteria have been shown to be located on the pilus tip mediating cell adherence and invasion [15, 23]. Therefore, it is plausible that lack of surface exposure of the AP1-2b protein in *Δbp-2b* contributes to the decreased adhesion/invasion capacity of this mutant.

Remarkably, in previous epidemiological studies investigating neonatal invasive GBS strains from eight European countries [24], among 42 investigated CC17 isolates we observed a wider high expression of the pilus type 2b (93% of strains reacted with anti-BP2b specific antibodies in flow cytometry experiments) compared to pilus 1 (only 10% anti-BP1 reactive strains).

Overall, the data here reported point toward a more prominent role of pilus 2b than pilus 1 in ST-17 pathogenesis by enhancing cell invasion, bacteremia and spread into the Central Nervous System and confirm that this protein could be a therapeutic and prophylactic target against neonatal sepsis and meningitis caused by this GBS lineage.

## Conclusions

We studied both type 1 and type 2b pili in the GBS strain COH1 and demonstrated for the first time that only GBS type 2b pilus contributes to adherence to host cells and survival in blood, brain and lungs in a mouse meningitis model. The possibility that both pilus types contribute synergistically to infection caused by ST-17 strains will deserve further investigation in a double knockout mutant lacking both pilus types. Our analysis gives support to recent studies where GBS pilins have been identified as important virulence factors and potential vaccine candidates [11].

## Methods

### Bacterial strains, plasmids and growth conditions

Bacterial strains and plasmids used in this study are reported in Table 1. *S. agalactiae* strains were routinely grown in Todd-Hewitt broth or on Todd-Hewitt agar (Difco; BD, Franklin Lakes, NJ, USA) at 37 °C. *Escherichia coli* cells were grown in Luria-Bertani medium. When required, erythromycin (Em) 1 μg ml^−1^ and chloramphenicol (Cm) 10 μg ml^−1^ were added to the medium.

**Table 1:** Bacterial strains and plasmids used in this study

Strain or plasmid	Relevant characteristic(s)	Source or reference
*E. coli*		
DH5α	F- φ80lacZΔM15 Δ(lacZYA-argF) U169 recA1 endA1 hsdR17 (rk-, mk+) phoA supE44 λ- thi-1 gyrA96 relA1	Invitrogen
Mach1™-T1R	F- φ80(lacZ)ΔM15 ΔlacX74 hsdR(rK-mK+) ΔrecA1398 endA1 tonA	Invitrogen
*S. agalactiae*		
COH1	GBS wild type	Rosini et al.
*Δbp-1*	COH1 knockout (KO) deleted of the gene coding for the backbone protein of pilus 1 (BP-1 or GBS80)	Rosini et al.
*Δbp-2b*	COH1 knockout (KO) deleted of the gene coding for the backbone protein of pilus 2b (BP-2b)	This study
*Δbp-2b+*	Δbp-2b complemented with pAM401_ bp-2b	This study
Plasmids		
pJRS233	6.0 kb; ColE1 *ori*; temperature-sensitive *E. coli*-streptococcal shuttle vector	Perez-Casal et al.
pJRS233_ *bp-2b*	pJRS233-derived containing overlapping flanking sequences of *bp-2b* gene	This study
pAM401/*gbs80*P + T	11.5 kb; Cmr; ColE1 *ori; E. Coli*-streptococcal shuttle vector pAM401 containing promoter and terminator of *gbs80* gene	Rosini et al.
pAM401_ *bp-2b*	pAM401/gbs80P + T-derived containing entire *bp-2b* coding sequence	This study

Serotype III-ST17 COH1, a clinical isolate obtained from an infected newborn with sepsis [25], was used for the construction of the knockout (KO) mutant strains (Δ*bp-1* and Δ*bp-2b*) carrying in-frame deletions of the genes coding for the pilus 1 and 2b backbone proteins. These mutants were generated using Splicing by Overlap Extension PCR as described previously [10].

To generate the complemented strain Δ*bp-2b*+, the *bp-2b* gene (locus tag SAN_1518) was PCR amplified from COH1 strain genome (HG939456.1) and cloned into the *E. coli*-streptococcal shuttle vector pAM401/gbs80P_T, generating the pAM401_*bp-2b* plasmid. This construct was used to transform by electroporation the Δ*bp-2b* mutant [10].

### Antibodies

Pilus proteins (BP-1, AP1–1, BP-2b and AP1-2b) were expressed as His-tagged fusion proteins and purified by affinity chromatography, as reported previously [11].

Mouse monoclonal antibodies (mAbs) were generated by Areta International (Varese, Italy) using standard protocols. Briefly, B-cell hybridoma clones were isolated from spleen cells of immunized CD1 mice with the purified recombinant pilus proteins. Hybridoma clones were screened by enzyme-linked immunosorbent assay (ELISA). Positive clones were then tested for binding to the surface of GBS by flow cytometry. The selected mAbs were finally purified by protein G affinity chromatography.

### Immunoblot

For the preparation of total soluble proteins, mid-exponential-phase GBS were harvested by centrifugation, washed in phosphate-buffered saline (PBS) and suspended in 50 mM Tris-HCl (pH 6.8) containing mutanolysin (Sigma-Aldrich, St. Louis, MO, USA) and complete protease inhibitors (Roche, Basel, Switzerland). Cell suspensions were then incubated 2 h at 37 °C and lysed by freeze and thaw. Soluble proteins were separated from cellular debris by centrifugation at 15000 x g at 4 °C for 10 min, then fractionated by sodium dodecyl sulfate–polyacrylamide gel electrophoresis (SDS-PAGE) and transferred to nitrocellulose membranes using iBlot transfer (Dry blot system, Invitrogen). Membranes were probed with purified mAbs (1:1000 dilution), followed by a rabbit anti-mouse horseradish peroxidase-conjugated secondary antibody (Dako, Glostrup, Denmark). Bands were then visualized using an Opti-4CN substrate kit (Bio-Rad).

### Flow Cytometry

Mid-exponential phase bacterial cells were harvested, washed in PBS, suspended in PBS containing 0.08% (wt/vol) paraformaldehyde and incubated for 1 h at 37 °C. Fixed bacteria were then washed in PBS/1% BSA, and incubated at RT for 20 min in newborn calf serum (Sigma, St. Louis, MO). The cells were then incubated for 1 h at 4 °C with primary antibodies diluted 1:200 in dilution buffer (PBS, 0.1% [wt/vol] bovine serum albumin, 20% [vol/vol] newborn calf serum). Cells were washed in PBS–0.1% Bovine Serum Albumin and incubated for a further 1 h at 4 °C with a 1:100 dilution of R-phycoerythrin-conjugated F(ab)2 goat anti-mouse IgG (Jackson ImmunoResearch Laboratories, Inc.). After washing, cells were suspended in PBS and analyzed with a fluorescence activated cell sorting (FACS) CANTO II apparatus (Becton, Dickinson, Franklin Lakes, NJ) using FlowJo Software (Tree Star, Ashland, OR).

### Mouse model of meningitis and bacterial blood survival assays

The mouse model of hematogenous GBS meningitis used in this study was described previously [26]. Briefly, 8-week-old male CD-1 mice (Charles River Laboratories, Wilmington, MA, USA) were injected via tail vein (i.v.) with 1.5 × 10^8^ CFU of GBS. At the experimental endpoint (day 2 p.i.) blood, brain and lungs were collected upon euthanasia. Tissues were homogenized and homogenates as well as blood were plated on THB agar for enumeration of bacterial CFU.

For blood survival assays mid log phase GBS (4 × 10^3^ CFU) were added into 0.2 ml of blood isolated from mice and incubated at 37 °C with rotation. The number of viable bacteria was determined by plating serial dilutions every 30 min up to 7 h. Each GBS strain was run in triplicate for each time point.

### Assay for GBS intracellular survival in macrophages

The murine macrophage-like cell line J774 (J774 A.1; ATCC TIB 67) was grown and maintained in Dulbecco’s modified Eagle’s medium (DMEM) (Invitrogen Life Technologies, CA, USA) supplemented with 10% fetal calf serum and 5 mM glutamine (Sigma Chemical Co., MO, USA). Cell monolayers grown at confluence in 24-well NUNC tissue culture plates were inoculated with 1 × 10^6^ GBS CFU in DMEM (multiplicity of infection 10:1) followed by 2 h incubation. After infection the monolayers were washed with cold PBS to remove non-adherent bacteria and added with 100 μg ml − 1 Penicillin, 100 μg ml − 1 Streptomycin and 100 μg ml − 1 Gentamicin. Cultures were further incubated for 48 h. After 0, 2 h, 6 h, 24 h and 48 h post-antibiotic treatment monolayers were rinsed with PBS, macrophages were lysed with 1 ml of distilled water for 10 min and plated for quantification of cell associated GBS bacteria. The mean CFU +/− SD of two independent experiments performed in quadruplicate was reported.

### Cell adherence and invasion assays

Immortalized human brain microvascular endothelial cells (hBMEC) were kindly provided by professor Kwang Sik Kim at Johns Hopkins University and cultured in RPMI1640 containing 10% FBS, 10% Nu-serum and 1% nonessential amino acids. Human A549 lung carcinoma cell line was obtained from the American Type Culture Collection (ATCC CCL-185) and cultured in RPMI1640 containing 10% FBS, and 1% nonessential amino acids. Cell lines were maintained at 37 °C with 5% CO_2_.

Cellular adherence and invasion assays were performed in triplicate and repeated at least three times as previously reported [26]. Briefly, mid-log grown bacteria were added to confluent cell monolayers at a multiplicity of infection of 1. Total cell-associated GBS bacteria were recovered after 30 min incubation, while intracellular GBS were recovered after 2-h infection and 2-h incubation with antibiotics to kill all extracellular bacteria. Bacteria were quantified by serial dilution plating on THA. Total cell-associated and intracellular GBS was calculated as (recovered colony forming units CFU/initial inoculum CFU) ×100%.

### Statistical analysis

GraphPad Prism Software was used for statistical analysis. Differences in CFU number measured in blood, brain and lung of mice infected with WT or mutant strains were analyzed by Kruskal-Wallis test followed by Dunn’s multiple comparisons test. The significance of differences in cell bacterial adherence and invasion to cells was determined using ONE-way ANOVA following by Tukey’s multiple comparisons test. Differences between WT and *Δbp-2b* survival in macrophages were assessed by non-parametric Mann-Withney test. Differences in growth kinetics in rich medium or in blood were assessed by 2-way ANOVA test.

## Abbreviations

AP: Ancillary protein
BBB: Blood-brain barrier
BP: Backbone protein
CC: Clonal complex
CFU: Colony forming units
ELISA: Enzyme-Linked immunosorbent assay
EOD: Early onset disease
GBS: Group B Streptococcus
HBMEC: human brain microvascular endothelial cells
KO: Knockout
LOD: Late onset disease
MAb: Mouse monoclonal antibody
PBS: Phosphate-buffered saline
SDS-PAGE: Sodium dodecyl sulfate–polyacrylamide gel electrophoresis
ST: Sequence type
WT: Wild-type
